# Internet addiction detection rate among college students in the People’s Republic of China: a meta-analysis

**DOI:** 10.1186/s13034-018-0231-6

**Published:** 2018-05-25

**Authors:** Yao-jun Shao, Tong Zheng, Yan-qiu Wang, Ling Liu, Yan Chen, Ying-shui Yao

**Affiliations:** Faculty of Epidemiology and Statistics, School of Public Health, Wannan Medical College, 22 Wenchang West Road, Yijiang District, Wuhu, 241002 Anhui People’s Republic of China

**Keywords:** China, College students, Internet addiction, Meta-analysis, Prevalence

## Abstract

**Background:**

With the development of economy and technology, the Internet is becoming more and more popular. Internet addiction has gradually become a serious issue in public health worldwide. The number of Internet users in China has reached 731 million, with an estimated 24 million adolescents determined as having Internet addiction. In this meta-analysis, we attempted to estimate the prevalence of Internet addiction among College Students in the People’s Republic of China in order to improve the mental health level of college students and provide evidence for the prevention of Internet addiction.

**Methods:**

Eligible articles about the prevalence of Internet addiction among college students in China published between 2006 and 2017 were retrieved from online Chinese periodicals, the full-text databases of Wan Fang, VIP, and the Chinese National Knowledge Infrastructure, as well as PubMed. Stata 11.0 was used to perform the analyses.

**Results:**

A total of 26 papers were included in the analyses. The overall sample size was 38,245, with 4573 diagnosed with Internet addiction. The pooled detection rate of Internet addiction was 11% (95% confidence interval [CI] 9–13%) among college students in China. The detection rate was higher in male students (16%) than female students (8%). The Internet addiction detection rate was 11% (95% CI 8–14%) in southern areas, 11% (95% CI 7–14%) in northern areas, 13% (95% CI 8–18%) in eastern areas and 9% (95% CI 8–11%) in the mid-western areas. According to different scales, the Internet addiction detection rate was 11% (95% CI 8–15%) using the Young scale and 9% (95% CI 6–11%) using the Chen scale respectively. Cumulative meta analysis showed that the detection rate had a slight upward trend and gradually stabilized in the last 3 years.

**Conclusion:**

The pooled Internet addiction detection rate of Chinese college students in out study was 11%, which is higher than in some other countries and strongly demonstrates a worrisome situation. Effective measures should be taken to prevent further Internet addiction and improve the current situation.

## Background

Internet addiction can be defined as overuse of the Internet leading to impairment of an individual’s psychological state (both mental and emotional), as well as their scholastic or occupational and social interactions [1]. Its symptoms generally include preoccupation, loss of control, high tolerance, withdrawal, craving, impairment of function and a reduction in the ability to make decision [2]. The prevalence of Internet addiction in American college students is 12% and the Internet addiction rate of Iranian medical students is 10.8% [3, 4]. Worse yet, studies have shown that the rate of Internet addiction in Serbian schoolchildren is 18.7% [5]. In China, as well as worldwide, Internet addiction is a significant growing health problem in college students which is harmful to their physical and mental health. According to a survey conducted by the China Internet Network Information Center, the number of Internet users in China has reached 731 million, which equals the total population in Europe. There is no doubt that the Internet has brought us a lot of benefits. The Internet provides young people with good conditions for learning and strengthen the communication between young people. It is necessary for students to learn how to use the Internet. Internet tools can be effectively applied in school education, specifically in areas of lectures, assignments, real-time procedure demonstration, class discussion, and interaction with teachers. Internet can also realize the sharing of learning resources. So it is useful to integrate this learning modality with the traditional mode of teaching through a well thought out curriculum modification [6]. Besides, Internet has changed the way people socialize and it has become a medium for disease prevention and health promotion. Because young people are able to participate in a growing numbers of online communities providing support and advice for health care. A study of disturbed adolescents found that computer-mediated communication diminished certain traditional gender differences in group communication [7, 8]. However, the disadvantages caused by the Internet cannot be ignored. Internet addiction brings a lot of risks to society. Firstly, it makes people spend more time on Internet games and reduce normal social activities [9]. Secondly, there is a lot of unhealthy information on the Internet, such as pornography, violence and so on, which can affect people’s mental health. The current findings suggest that adolescents with Internet addiction seem to have more aggressive dispositions than non-Internet addicted adolescents [10]. Finally, Internet addiction leads to lack of sleep, vision disturbances and decline in work efficiency, which are detrimental to our physical health [11]. Therefore, it is crucial for us to investigate the prevalence of Internet addiction among Chinese college students in order to provide epidemiological information to better understand and tackle this problem.

To the best of our knowledge, currently there is no consensus on the standard for the diagnosis and identification of Internet addiction disorder. Young’s Internet Addiction Diagnostic Questionnaire (YDQ) was compiled in 1983. A respondent who answers yes to five or more of the eight questions is diagnosed as addiction Internet user. This questionnaire was further developed in 1998 by Young in order to incorporate the DSM-IV pathologic gambling criteria [12]. This 20-item scale, with its score ranging from 0 to 100, is widely used in diagnosing Internet addiction. Respondent with the total score ranging from 50 to 79 is considered moderate Internet user and 80–100 as severe Internet user with serious problems in Internet use. Previous studies have demonstrated that the scale has a high reliability and validity [12]. To take group differences into account, the Chen Internet Addiction Scale (CIAS) is used to measure the extent of Internet addiction. There are 26 items in the CIAS, and an individual with a score of 68 or more is assessed as Internet addiction [13]. A revision of the CIAS with 19 questions was assembled by Bai in 2005, which divides Internet addiction into three level: normal (from 19 to 45), moderate (from 46 to 53) and excessive (above 53). These scales have been gradually used in Internet addiction research in China.

A lot of in-depth research on drug addiction has been explored, such as the epigenetic mechanisms of drug addiction. Unlike drug addiction, the influence of Internet addiction has been underestimated and few studies explore the mechanisms of it [14]. With the Internet addiction becoming more and more serious, relevant government departments begin to pay more attention to the effects of Internet addiction on teenagers and college students. Since their physical and mental development is not yet mature, their abilities of self regulation and control remain to be improved [15, 16]. In this meta-analysis, we attempted to investigate the prevalence of Internet addiction among college students in the People’s Republic of China in order to provide epidemiological evidence for the prevention of Internet addiction and finally improve the mental health level of college students.

## Methods

### Search strategy

Articles related to Internet addiction between 2006 and 2017 were retrieved from the Chinese periodical databases of Chinese National Knowledge Infrastructure, VIP and WanFang and from PubMed. We searched the following keywords: “Internet addition“, “college students/university students”, “detection rate” and “China”. Languages were restricted to English and Chinese. In addition, relevant articles were manually searched.

### Selection criteria

Inclusion criteria included: the research objects are full-time Chinese college students or vocational college students who are 18–25 years old; published between 2006 and 2017; using random sampling method; discussion of the Internet addiction detection rate in Chinese college students with reliable and clear statistics; Internet addiction is defined clearly and Internet addiction related questionnaire was adopted. CIAS has a Cronbach’s alpha of 0.95, and YDQ has a Cronbach’s alpha of 0.93 as well as a good test–retest reliability (*r *= 0.85) [3, 13]; high quality articles have priority among the same subjects (For articles in which the same subjects were included in different publications, only the most recent or complete study was included). Exclusion criteria consisted of: articles unrelated to the purpose of the study; valid data cannot be extracted from the study; data is incomplete or repeated publication.

### Literature screening and quality assessment

According to selection criteria, data extraction was completed independently by two researchers. Disagreements were solved by discussion or a third reviewer. For missing information, we contacted the correspondent authors for completed data. The following information was extracted from the literature: first author, year of publication, investigation time and area, sampling method, sample size, gender composition, and the scale used for Internet addition. Evaluation tools recommended by Agency for Healthcare Research and Quality (AHRQ) were used to measure the quality of research [17].

### Statistical analysis

Stata 11.0 software was used for the analysis. According to the results of heterogeneity test, the random effects model was used. Subgroup analyses, cumulative meta-analysis and chart description were also performed. Begg’s and Egger’s test were applied to examine publication bias [18].

## Results

### Basic information and quality assessment

**Fig. 1:**
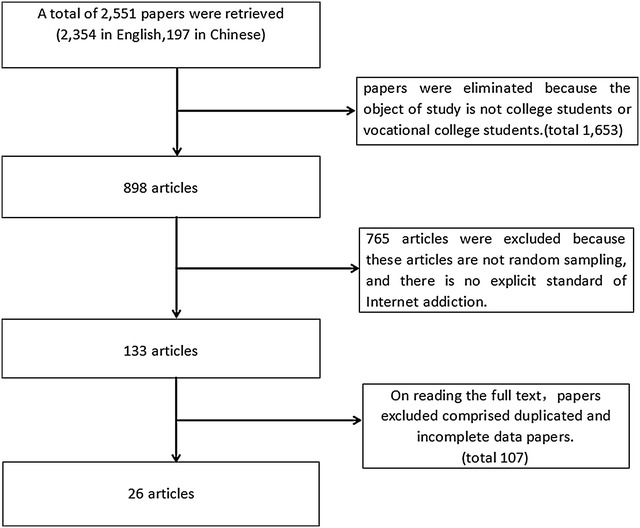
Flow chart of literature search

A total of 2551 articles were initially retrieved from the online Chinese periodical full-text Chinese National Knowledge Infrastructure (n = 2033), VIP (n = 214), Wan Fang (n = 107) databases, and from PubMed (n = 197). By reading the title 1653 articles were eliminated since the object of study was not college students or vocational college students, most of these articles instead are devoted to the study of middle school students. After quality evaluation, 765 articles were further excluded. Of these, 157 articles did not mention sampling method and 319 articles did not use random sampling method. Another 289 articles had no explicit standard of Internet addiction or a clear definition of Internet addiction. In addition, 107 articles were removed after reading the full text because of lacking necessary data or containing incomplete data. Finally, 26 articles were included. Figure 1 shows the literature search process. The total sample size was 38,245 college students, the largest sample was 4866, and the smallest was 434. 4573 students were diagnosed as Internet addiction. Main characteristics of the included 26 eligible articles are shown in Table 1.

**Table 1:** Main characteristics of studies showing Internet addiction detection rates among college students in China

References	Years	District	Prevalence of Internet addiction (%)			Scale	Subject
			Total (IA/sample size)	Male (IA/sample size)	Female (IA/sample size)		
Yao et al. [34]	2006	Wuhu	12.9 (260/2010)	16.1 (229/1427)	5.3 (31/583)	Young scale	College student
Feng et al. [35]	2007	Guizhou	8.4 (126/1497)	11.1 (75/675)	6.2 (51/822)	Young scale	College student
Wang et al. [36]	2007	Dalian	7.3 (70/954)			Young scale	College student
Chen and Fan [37]	2008	Hefei	4 (28/705)	6 (22/364)	1.8 (6/341)	Young scale	College student
Gao et al. [38]	2008	Changchun	7.8 (96/1227)	12.7 (51/403)	5.5 (45/824)	Young scale	College student
Zhang et al. [39]	2009	Ningbo	11.7 (119/1014)	18.1 (108/597)	2.6 (11/417)	Young scale	College student
Liu et al. [40]	2009	Wuhan	4.6 (20/434)	7.3 (15/207)	2.2 (5/227)	Young scale	College student
Gao and Ma [41]	2009	Hangzhou	11.9 (81/683)	16.7 (51/306)	8 (30/377)	Young scale	College student
Ju-Yu Yen et al. [42]	2009	Taiwan	12.3 (246/1992)	19.1 (111/581)	9.6 (135/1411)	CIAS	College student
Zhou et al. [43]	2010	Daqing	10.8 (85/787)	18.6 (44/237)	7.5 (41/500)	Young scale	College student
Zhang et al. [44]	2011	Dali	10.4 (100/965)	13.6 (46/338)	8.6 (54/627)	Young scale	College student
Zhao et al. [45]	2012	Lanzhou	11.1 (200/1807)	13.5 (125/926)	8.5 (75/881)	Young scale	College student
Chen et al. [46]	2012	Wuhan	6.8 (32/470)	11.1 (20/181)	4.2 (12/289)	CIAS	College student
Zhang et al. [47]	2013	Jinan	5.5 (52/853)	11.4 (32/280)	3.5 (20/573)	CIAS	College student
Luo et al. [25]	2014	Shandong	4.5 (46/1026)	8.1 (31/384)	2.3 (15/642)	Young scale	College student
Zhang [48]	2014	Xinjiang	8.7 (90/1037)			CIAS	College student
Zhou et al. [49]	2014	Wuxi	12.8 (621/4866)	15.9 (338/2122)	10.3 (283/2744)	Young scale	College student
Luo and Zhu [50]	2015	Jiangxi	7.2 (39/545)	16 (19/119)	4.7 (20/426)	Young scale	College student
Wang et al. [51]	2015	Hainan	33.4 (781/2341)	38.4 (312/812)	30.7 (469/1529)	Young scale	College student
Zhang et al. [52]	2015	Nantong	10.8 (450/4168)	12.2 (185/1515)	10 (265/2653)	CDC standard	College student
Zhou et al. [24]	2015	Yan’an	19.5 (117/601)	27.6 (48/174)	16.2 (69/427)	Young scale	College student
Cong et al. [53]	2016	Yantai	43.9 (249/567)	55.2 (95/172)	39 (154/395)	Young scale	College student
Chi et al. [54]	2016	Hefei	15.2 (178/1173)			Young scale	College student
Chen et al. [55]	2016	Hebei	9.6 (234/2451)	13.5 (162/1204)	5.8 (72/1247)	CIAS	College student
Wu et al. [56]	2017	Taishan	6.7 (93/1385)	9.1 (36/394)	5.8 (57/991)	Young scale	College student
Li et al. [57]	2017	Henan	6 (160/2687)	8.6 (93/1087)	4.2 (67/1600)	Young scale	College student

*CDC* Chinese Center for Disease Control and Prevention, *CIAS* Chen Internet Addiction Scale, *IA* Internet addiction, *Young* Young Internet Addiction Scale

### Meta-analysis of Internet addiction detection rates in college students in the People’s Republic of China

**Fig. 2:**
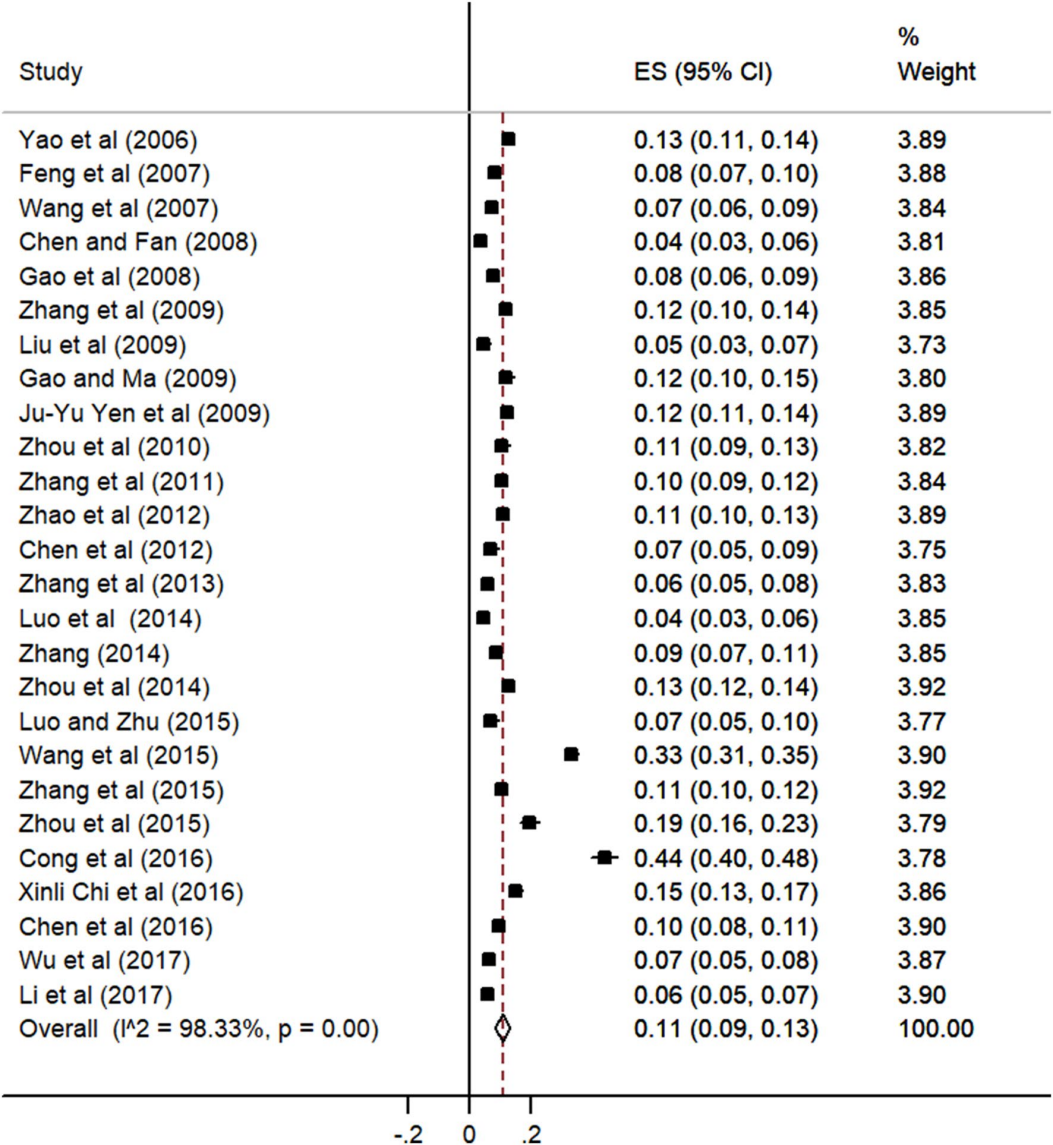
Forest plot of Internet addiction prevalence and confidence intervals

A total of 26 articles reported Internet addiction detection rate among college students in China. Heterogeneity test showed a result of I^2^ = 0.983, indicating heterogeneous among studies. Therefore random-effects model was chosen. The pooled prevalence of Internet addiction in Chinese college students was 11% (95% confidence interval [CI] 9–13%), the result is shown by the forest plots in Fig. 2.

### Subgroup analyses

**Fig. 3:**
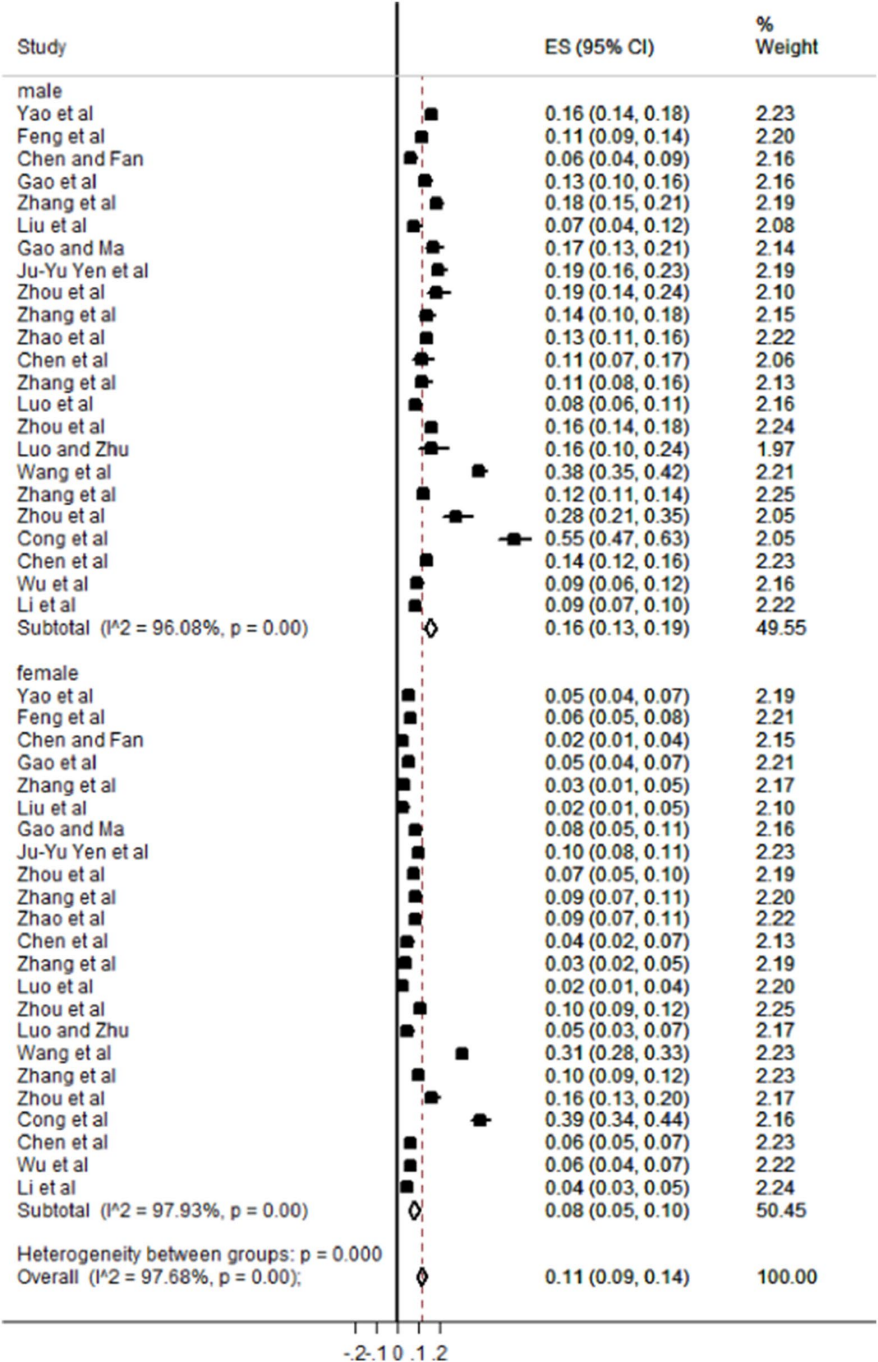
Forest plot of subgroup analysis based on gender

In order to find the source of heterogeneity, subgroup analysis was performed according to stratum of gender, region, and scale. The result of subgroup analyses were presented in Table 2. There is a statistically significant difference of the Internet addiction detection rates between male students and female students (P < 0.05). The mean prevalence of Internet addiction was 16% (95% CI 13–19%) for male students and 8% (95% CI 5–10%) for female students respectively (Fig. 3). The Internet addiction detection rate was 11% (95% CI 8–14%) in southern areas, 11% (95% CI 7–14%) in northern areas, 13% (95% CI 8–18%) in eastern areas and 9% (95% CI 8–11%) in the mid-western areas. According to different scales, the Internet addiction detection rate was 11% (95% CI 8–15%) using the Young scale and 9% (95% CI 6–11%) using the Chen scale.

**Table 2:** Mean prevalence of Internet addiction among college students in different subgroups

	Gender		District distribution				Scale	
	Male	Female	South	North	East	Mid-west	Young	CIAS
Study number	23	23	14	12	11	15	20	5
Prevalence (%)	16	8	11	11	13	9	11	9
95% CI (%)	13–19	5–10	8–14	7–14	8–18	8–11	8–15	6–11
Heterogeneity (I^2^)	0.961	0.979	0.984	0.981	0.991	0.939	0.987	0.902

North and South are divided by Qinling Mountains–Huaihe River Line. East and mid-west are divided by economic development level. One paper which uses CDC standard do not sort by scale

*CI* confidence interval, *CIAS* Chen Internet Addiction Scale, *Young* Young Internet Addiction Scale

### Cumulative meta-analysis

**Fig. 4:**
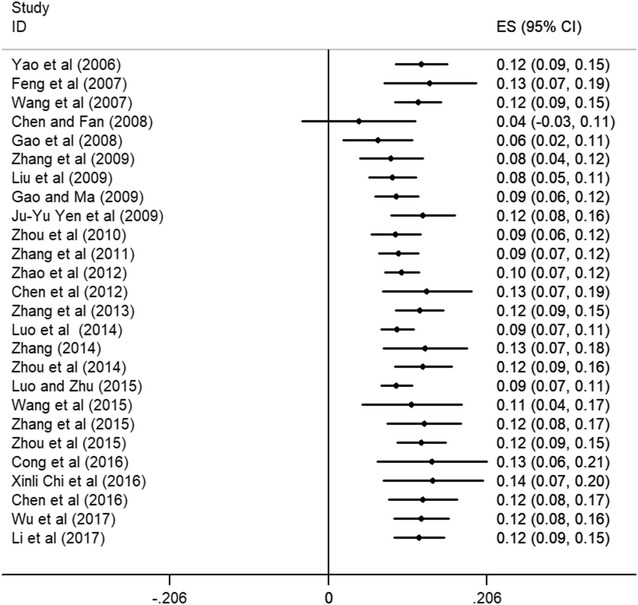
Cumulative meta-analysis based on year

Cumulative meta-analysis was carried out for the detection rate based on year and sample size. The detection rate had a slight upward trend and gradually stabilized around 12% in the past 3 years as shown in Fig. 4. As for sample size,the detection rate grew more stable with the increase of sample size, also reaching 12%.

### Publication bias

**Fig. 5:**
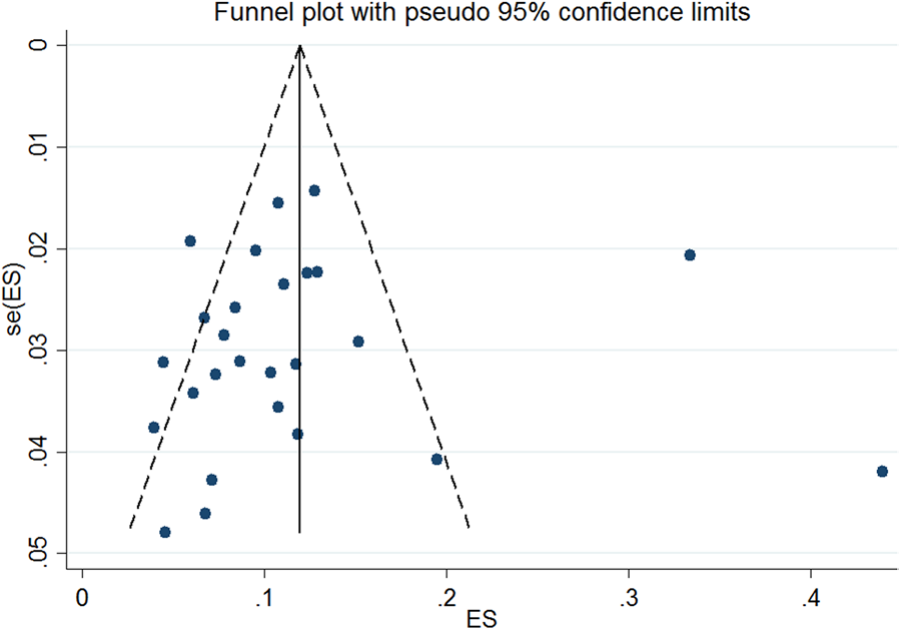
Funnel plot of overall prevalence

Publication bias was assessed using the funnel plots (Fig. 5) [19]. Begg (z = 0.44, P = 0.659) and Egger test (t = − 0.31, P = 0.761) results suggested a low possibility of publication bias.

## Discussion

The Internet has become an indispensable part of our lives, providing us more convenience. We rely heavily on the Internet, which also brings serious negative effects, such as game addiction. The influence of Internet addiction on college students as a special group has become a hot issue in public health. In this meta-analysis, 26 articles related to Internet addiction published between 2006 and 2017 were retrieved from databases based on our strict inclusion and exclusion criteria. As shown in Table 1, Internet addiction detection rates among college students in China varied widely from 4 to 43.9%, possibly due to the sample sizes, economic development differences and time of investigation. Economic is more developed in eastern coastal areas of China than that in other areas, which results in earlier Internet touching among young people in east China. Currently, Internet has gradually become popular in east China. Since few people have been in contact with computer decades ago, low rate of Internet addiction was reported at that time. Our study reflects the general characteristics of Internet addiction prevalence among Chinese college students. A previous study proved that the rate of Internet addiction among teenagers in the world is 10% [20]. In our study, the pooled prevalence of Internet addiction in Chinese college students is 11% (95% CI 9–13%), which is similar to many studies conducted in China but different from studies conducted abroad. Compared with other countries, the detection rate in China is higher than Japan [21] (3.7%) and Italy [2] (4.3%), but similar to Pakistan [22] (16.7%), Chile [12] (11.5%) and Turkey [23] (9.7%).

After subgroup analyses, we find that Internet addiction has different effects on male and female students, with higher detection rates in male students (16%) than in female students (8%). It may be explained by the differences in coping styles when facing life stress or negative life events. Male students tend to solve problems on their own and are reluctant to communicate with others or ask for help, leading to the low utilization of social support [24]. Some studies report that males are more sensitive to the Internet than females [25]. Compared with females, online games are more attractive to males who have a greater breadth of Internet use and more time surfing on Internet [26]. The above factors may contribute to a higher detection rate in male students. In terms of the regional factor, the Internet addiction detection rate was 11% in northern and southern areas in China. A higher detection rate was seen in the eastern areas as compared with mid-west. The regional difference could be caused by uneven economic development between eastern and mid-west areas, with more popularity of the Internet in the eastern areas attracting more college students. Our findings show that the Internet addiction detection rate using the Young scale was higher than that using the Chen scale. These two scales are widely used in the measurement of Internet addiction, and further research should be made to compare and evaluate the two scales.

According to the results of cumulative meta-analysis, the Internet addiction detection rate of Chinese college students has increased slowly since 2008 and gradually stabilized around 12% in the past 3 years. This shows that the Internet addiction has become an increasingly serious problem which can lead to many negative effects on college students, including physical and mental health. Internet addicts are more obvious in obsessive-compulsion, interpersonal sensitivity, depression, anxiety, hostility and other problems. Their mental health level is lower because they are addicted to the Internet for a long time which results in the lack of interpersonal communication, which in itself is a risk factor for mental illness [24]. Furthermore, Internet addiction can also cause many somatic diseases such as neurasthenia, decreased vision, lack of concentration, and sleep disorder. Worst of all, Internet addiction can cause conduct disorder, inducing teenagers to play truant even crime. This study still has limitations: the diagnosis of Internet addiction is only measured by self report, with no clinical assessment of disability or other sources of information. It may have an impact on the integrity of the information collection and the results accuracy. Thus, we increase the assessment of other information in further research.

## Conclusion

According to the research, the mean prevalence of Internet addiction in Chinese college students was 11%. Boys (16%) have a higher rate of Internet addiction than girls (8%). Given the rising Internet addiction rates among college students in China, effective and practical intervention measures should be taken. On one hand, government should strengthen the supervision of the Internet and provide legal protection in order to reduce the harm to college students. For example, no Internet cafes is allowed to be open within 200 meters in school, the opening hours of Internet cafes must be limited to between 8 a.m. and midnight, and an anti-addiction system should be established to limit the time spending on online games [27]. On the other hand, the university should encourage students to participate in more social activities and athletic sports [28]. In addition, parents should increase communication with their kids and spend more time relieving their inner troubles as well as understanding their needs [29–31]. In my opinion, it is also important to take measures to educate society about the dangers of Internet addiction. First of all, some measures need to be taken in communities and schools where more lectures on Internet addiction can be carried out [32, 33]. Schools and communities must guide students to use the Internet when they enter school and build a good way to communicate with their parents. Secondly, parents need to set up an Internet usage plan for children to make them know the seriousness of the Internet addiction [30]. Finally, the mass media can also organize more social activities such as Internet knowledge competition and make a documentary about Internet addiction so that people learn more about the dangers of Internet addiction. The most important factor is to help people form a reasonable understanding of Internet addiction and change unhealthy lifestyles. It is very necessary for us to pay more attention to the social education of Internet addiction in future studies. Only in this way, Internet addiction will lessen and young people will have a healthy environment to grow up.

## Abbreviations

CI: confidence interval
CIAS: Chen Internet Addiction Scale
Young: Young Internet Addiction Scale
CDC: Chinese Center for Disease Control and Prevention
IA: Internet addiction
VI: VIP Database for Chinese Technical Periodicals
DSM-IV: Diagnostic and Statistical Manual of Mental Disorders-Fourth Edition
AHR: Agency for Healthcare Research and Quality
YDQ: Young’s Internet Addiction Diagnostic Questionnaire
